# Religious Belief-Related Factors Enhance the Impact of Soundscapes in Han Chinese Buddhist Temples on Mental Health

**DOI:** 10.3389/fpsyg.2021.774689

**Published:** 2022-01-26

**Authors:** Dongxu Zhang, Chunxiao Kong, Mei Zhang, Jian Kang

**Affiliations:** ^1^School of Architecture and Urban Planning, Guangzhou University, Guangzhou, China; ^2^School of Architecture and Urban Planning, Shenyang Jianzhu University, Shenyang, China; ^3^School of East Asian Studies, The University of Sheffield, Sheffield, United Kingdom; ^4^Institute for Environmental Design and Engineering, The Bartlett, University College London, London, United Kingdom

**Keywords:** Han Chinese Buddhist temple, soundscape, evaluation, mental health, religious belief-related factors

## Abstract

In contemporary society, mental health issues have received increasing attention. Moreover, how people perceive the acoustic environment affects mental health. In religious places, the unique religious soundscape, composed of the acoustic environment and sounds, has an obvious effect on mental health. In China, Han Chinese Buddhism has a long history and is currently the religion with the largest number of believers. The soundscape of temples has always been an important component of creating a Buddhist atmosphere. For this study, questionnaires were distributed to believers and tourists inside and outside several well-known Han Chinese Buddhist temples in China to analyse the relationship between evaluations of temple soundscapes (including the overall acoustic environment and preferences for typical sounds) and mental health and the role of religious belief-related factors in this relationship. The results indicated that for the respondents, the overall acoustic environment of Buddhist temples was significantly correlated with mental health and that a preference for three sounds in Buddhist temples, i.e., bells, wind chimes and chanting sounds, was significantly correlated with mental health. Among religious belief-related factors, attitudes toward Buddhist thought, frequency of temple visitation and purpose for visiting temples can affect the correlation between personal evaluations of temple soundscapes and mental health. For people who partially believe in Buddhist thought, people who visit Buddhist temples twice or less per year, or people who visit temples for tourism purposes, the correlations between evaluations of the overall acoustic environment and mental health are higher than for people without these religious characteristics. For people who fully believe in Buddhist thought or who visit temples neither to worship Buddha nor for tourism purposes, the correlations between the preferences for bells and wind chimes and mental health are higher than for people without these religious characteristics. For people who partially believe in Buddhist thought, the correlation between the preference for chanting and mental health is higher than for people with other attitudes toward Buddhist thought.

## Introduction

The concept of soundscapes was first proposed by the Finnish geographer Granö in 1929. In the 1960s and 1970s, the Canadian composer Schafer conducted research by treating soundscapes as an artistic concept. The ISO 12913-1 standard defines the term soundscape as “an acoustic environment as perceived or experienced and/or understood by a person or people, in context” (ISO, 2014). The study of soundscapes has become a widely researched topic in academia, and related interdisciplinary research has continuously emerged (Kang et al., 2016; Jahani et al., 2021).

Human mental health is an important component of health. The World Health Organization (WHO) proposes that health is not only the absence of disease or frailty but also a state of physical, psychological, and social integrity. However, many people in modern society have mental health issues. According to the WHO, the average life expectancy of people with mental disorders is approximately 20 years shorter than that of people with normal mental health. In 2017, 264 million people worldwide experienced anxiety. Based on data released by the Bureau of Disease Control and Prevention of the National Health Commission of China, the proportion of people with mental disorders in the Chinese population is as high as 17.5% (Www.cir.cn, 2019). Therefore, there is an urgent social need to explore ways to prevent and treat mental health problems. Scholars have conducted many studies on mental health, some of which have focused on the relationship between mental health and soundscapes or acoustic environments. These results showed that the quiet rural soundscape might benefit the general mental health of the population through its potential for psychological restoration (De Coensel and Botteldooren, 2006). The experience of quietness supports health, resulting in a lower degree of annoyance and contributing to physiological and psychological well-being (Öhrström et al., 2006). The experience of pleasant soundscapes facilitates faster recovery from stress (Medvedev et al., 2015). Noise exposure or excessive reverberation affects the well-being of children at school in their early childhood (Astolfi et al., 2019). Positive experiences of natural sound help subjects recovering from stress-related mental disorders (Cerwén et al., 2016). Better health conditions are associated with greater satisfaction with everyday soundscape experience (Booi and Van den Berg, 2012). The association between the physiological responses and the well-founded psychological components of the soundscape has also been explored (Erfanian et al., 2019). A systematic review was performed using three major scientific databases, and the findings revealed that regardless of the scale, statistically significant associations existed between positive soundscape perceptual constructs and health benefits (Aletta et al., 2018). For the relationship between noise and mental health, a narrative review documented the role of noise in clinical environments and its deleterious effects with a particular focus on mental health care (Brown et al., 2015). The association between mental well-being and the physical environment, such as neighbour noise, has been confirmed (Guite et al., 2006), and research (from 2003 to 2008) on the mental health effects of noise in adults and children has been summarised (Van Kamp and Davies, 2008). The results from international surveys suggested that long-term noise exposure was associated with mental health problems such as anxiety and depression (Stansfeld et al., 2000; Stansfeld and Matheson, 2003).

Previous studies have shown that individual characteristics could affect the relationship between the acoustic environment and mental health. For example, the correlation between the score on the mental health scale and the sound environment was affected by the respondent characteristics, such as gender, age, education, and noise sensitivity (Van Kempen et al., 2011); neighbourhood noise had different effects on the mental health of children, adults, and older people (Niemann et al., 2006). A report in a Turkish suggested that among students over 14 years old, men showed a higher annoyance in noise pollution (Anilan, 2014).

Soundscapes in religious places have been one of the important research objects in academia in recent years, including ancient English church music and soundscape maps (Burgess and Wathey, 2000), the effect of church bells on the soundscapes of early modern European towns (Garrioch, 2003), soundscapes in Islamic mosques in the Netherlands (Arab, 2017), and soundscapes in Han Chinese Buddhist temples (Zhang et al., 2016). Regarding the relationship between mental health and religious soundscapes or music, research has shown that the pleasantness of soundscapes in the two religious precincts in Korea significantly affects the perception of tranquillity, and the evaluation of tranquillity includes the degree of stress and restlessness (Jeon et al., 2014). Religious songs are an important form of religious expression important to the mental health of older African Americans (Hamilton et al., 2013). The frequency of listening to religious music among older Americans is associated with a decrease in death anxiety and increases in life satisfaction, self-esteem, and a sense of control (Bradshaw et al., 2015). Quran recitation could be introduced as an effective treatment for physical and mental diseases (Jafari et al., 2016). In terms of the relationship between religious beliefs and mental health, although some studies have demonstrated that the two are correlated (Koenig et al., 2001; Mueller et al., 2001; Maselko and Buka, 2008), there are relatively few studies on the relationship between Han Chinese Buddhism and mental health.

In summary, soundscapes and acoustic environments can affect mental health. In Western society, the relationship between the soundscapes or acoustic environments of religious sites and mental health has received attention. In China, the world’s most populous country, Han Buddhism has the largest number of religious believers, but there is nonetheless a lack of research on the relationship between the soundscapes of Han Chinese Buddhist temples and mental health and whether the relationship is influenced by religious belief-related factors. Recently, an increasing amount of attention has been given to the mental health of Chinese people; therefore, it is necessary to conduct research on this topic. As an important concept in statistics, moderation refers to whether the influence of variable X on variable Y can be moderated by variable Z. This paper aims to analyse the relationship between people’s evaluations of the soundscape in Han Chinese Buddhist temples and their mental health, as well as the moderator effects of religious belief-related factors on this relationship. First, questionnaires regarding temple soundscapes were distributed inside and outside Han Chinese Buddhist temples. The Kessler Psychological Distress Scale 10-item (K10) was used in the questionnaire to investigate respondents’ mental health. Based on the questionnaire results and the relationship between evaluations of Han Chinese Buddhist temple soundscapes and mental health, the influence of religious belief-related factors on the correlation between evaluations of the overall acoustic environment of Buddhist temples and mental health and the correlation between respondents’ sound preferences in Buddhist temples and mental health were analysed.

## Materials and Methods

### Questionnaire Design

This study used a questionnaire survey composed of four parts. The first part obtained basic demographic information from respondents, including gender, age, education level and occupation. The second part included items related to Buddhist beliefs, including attitudes toward Buddhist thought, the annual frequency of attending religious activities or visiting Buddhist temples and the purpose of visiting Buddhist temples. The third part was used to evaluate Buddhist temple soundscapes, including the importance of the acoustic environment of temples; respondents’ evaluations of the quietness, comfort and harmony of the overall acoustic environment of temples; and respondents’ preferences regarding six typical sounds in Han Chinese Buddhist temples (Buddhist instruments, chanting, bells, drums, background electronic Buddhist music and wind chimes). According to a previous questionnaire, among the sounds that can be heard in temples, respondents considered these six sounds to occur frequently and to be characteristic of religious places.

In the fourth part of the questionnaire, respondents’ mental health was assessed with the K10 scale, which contains 10-items divided among four factors: nervousness, restlessness, fatigue, and negative affect (see Table 1). Among them, nervousness, restlessness, and fatigue were assessed with two items each, while negative affect was assessed with four items, separately targeting the occurrence frequency of non-specific mental health-related symptoms, such as anxiety and stress, experienced by the respondents in the past 4 weeks. The occurrence frequency of each item was divided into the following five levels, each of which was scored from 1 to 5 points: all of the time, most of the time, some of the time, a little of the time and none of the time (Zhou et al., 2008). The scores for the 10-items (total possible score of 50 points) were summed, and the individual’s mental health status was divided into four levels based on the score: 10–30 points (very poor mental health), 31–38 points (poor mental health), 39–44 points (good mental health), and 45–50 points (very good mental health) (Kessler et al., 2002).

**TABLE 1:** Corresponding evaluation content of Kessler Psychological Distress Scale 10-item.

Evaluation content	Corresponding item
Nervousness	Did you feel nervous?
	Did you feel so nervous that nothing could calm you down?
Restlessness	Did you feel restless or fidgety?
	Did you feel so restless that you could not sit still?
Fatigue	Did you feel tired out for no good reason?
	Did you feel that everything was an effort?
Negative affect	Did you feel hopeless?
	Did you feel depressed?
	Did you feel so sad that nothing could cheer you up?
	Did you feel worthless?

### Determination of the Number of Questionnaires

The number of questionnaires in this study was determined based on previous studies (Zhang et al., 2018). First, 136 pilot questionnaires were tested before the formal questionnaires were distributed. The statistical results of these pilot questionnaires showed that the maximum standard deviation (SD) of the items for the acoustic environment and sounds was 1.135.

The maximum number of total questionnaires required was based on the empirical formula below (Du, 2010).


$$n={\left(\frac{u_{a/2}\,S}{d}\right)}^{2}$$

where n is the maximum sample size, ua/2 is a constant based on the confidence level, S is the estimate of the standard deviation, and d is the absolute limit of error.

In this study, the absolute limit of error “d” was set to 0.1, and the confidence level was set to 95%. Then, ua/2 = 1.96 and S was 1.135 in the test questionnaires, and the maximum sample size “n” was 495.

For this study, a final total of 521 questionnaires were distributed to and collected from tourists and believers in several well-known Han Chinese Buddhist temples. All respondents were clearly informed in advance about the purpose of the questionnaire survey and agreed to participate. After 22 invalid questionnaires were excluded, 499 valid questionnaires were eventually analysed. The maximum SD of the items in the formal questionnaire was 1.124 (less than 1.135 for the pilot questionnaire), indicating that the number of questionnaires distributed in this study met the requirements.

### Questionnaire Reliability and Validity

Reliability and validity analyses of questionnaires are a necessary step before data analysis. This study used SPSS software (version 25.0) for reliability and validity analyses of the results for the evaluations of overall acoustic environment and sound preferences (not including personal information and the K10 scale, which has been verified by numerous studies). The Cronbach coefficient for the evaluations of the overall acoustic environment was 0.803, indicating acceptable reliability of the data (Wu, 2010). Then, factor analysis was used to verify the construct validity of the questionnaire, that is, to test the extent to which the results of the questionnaire correctly verified the ideal assumption in the questionnaire design. Factor analysis was conducted on the results of the acoustic environment evaluation, and the results showed that the Kaiser-Meyer-Olkin (KMO) value was 0.735 and that the significance value for Bartlett’s test of sphericity was 0.000 (less than 0.05). These findings indicated that the data can be subjected to factor analysis (Du, 2010). Based on a characteristic root > 1, one principal component was obtained, and the cumulative contribution to all variables was 55.566% (>50%); therefore, the factor analysis results were acceptable (Wu, 2010). The reliability analysis of the results for sound preferences indicated that the Cronbach coefficient was 0.883. The factor analysis showed that the KMO value was 0.879 and that the significance value for Bartlett’s test of sphericity was 0.000 (less than 0.05), indicating that the data were suitable for factor analysis. Based on a characteristic root > 1, six principal components were obtained, the calculated results of the Cronbach coefficients for the six principal components were 0.857, 0.848, 0.849, 0.813, 0.832, and 0.851, respectively, and the cumulative contribution to all variables was 56.920% (>50%); therefore, the factor analysis results were acceptable (Wu, 2010), and the structural validity requirements were required.

### Determination of Calculation Methods

In this study, different calculation methods and indicators were selected based on the types of variables (see Table 2).

**TABLE 2:** The calculation method of independent and dependent variables.

Independent and dependent variables	Variable type	SPSS calculation approach	Index
Gender vs. mental health scores, attitude toward Buddhist thought, frequency of temple visitation, purpose	Dichotomous (nominal) variable/Continuous variable (Ordinal variable)	Independent-samples *t*-test	Mean difference
Purpose, occupation vs. mental health scores	Nominal variable/Continuous variable	Crosstabs	Phi and Cramer’s V
Age, education level, attitude toward Buddhist thought, frequency of temple visitation, evaluation of quietness (comfort and harmony), preferences for sounds vs. mental health scores	Ordinal variable/Continuous variable	Bivariate correlation	Spearman
Age, education level vs. attitude toward Buddhist thought, frequency of temple visitation	Ordinal variable/Ordinal variable	Crosstabs	Gamma

### Respondents’ Personal Characteristics

The basic demographic characteristics of the respondents in 499 questionnaires were collected and statistically analysed. Men and women accounted for 39.08% (195) and 60.92% (304) of the total respondents, respectively. The subjects were divided into three groups based on age: 29 years of age and younger, 30–44 years of age, and 45 years of age and older. These groups accounted for 35.47% (177), 38.28% (191), and 26.25% (131) of the respondents, respectively. In terms of education level, 11.42% (57) of the respondents had a junior high school education or below, 16.03% (80) had attended technical secondary school or high school, 53.71% (268) had attended vocational school or college, and 18.84% (94) had a master’s degree or above. In terms of occupation, survey respondents were divided as follows: students 18.04% (90) of the respondents, teachers 15.63% (78), technicians 11.82% (59), service personnel 7.62% (38), management personnel 7.41% (37), workers 7.01% (35), and other occupations 32.46% (162).

In terms of their attitudes toward Buddhist thought, 12.22% (61) of the respondents fully believed in Buddhist thought, 74.95% (374) partially believed in Buddhist thought, and 12.83% (64) had little or no faith in Buddhist thought. In terms of temple visiting frequency, 47.09% (235) of respondents visited the temple twice or less per year, 42.89% (214) visited the temple three to five times per year, and 10.02% (50) visited the temple more than five times per year. In terms of the purpose of visiting Buddhist temples, tourism accounted for 55.11% (275), worship accounted for 27.05% (135), and other purposes accounted for 17.84% (89).

## Results

The temple soundscape results from the questionnaire, including respondents’ evaluations of the overall acoustic environment of temples and their sound preferences, were analysed. Then, the mental health scores for the respondents were calculated, and correlation analysis was conducted for the temple soundscapes and mental health scores.

### Results of the Questionnaire Survey

**FIGURE 1:**
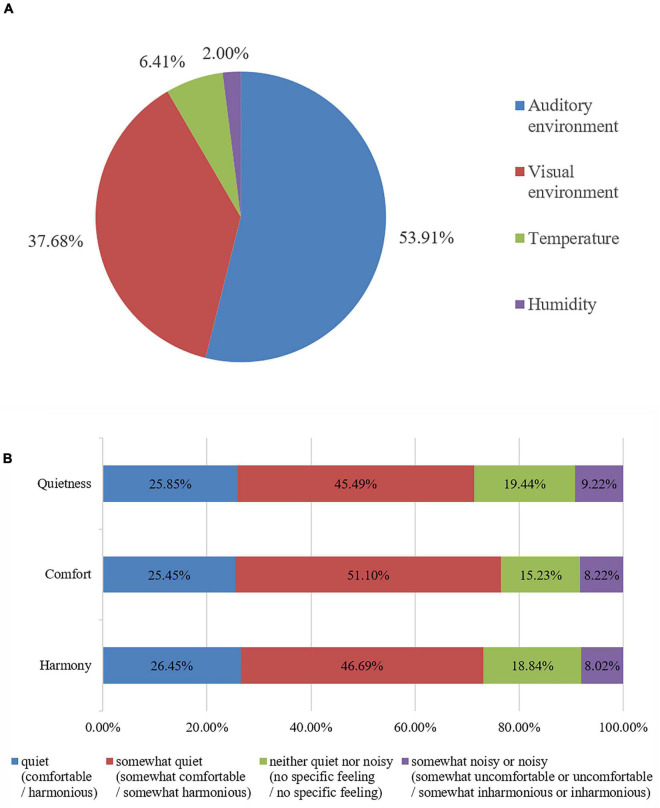
Distribution of the respondents’ environmental evaluations of the temple. **(A)** The first influencing factor; **(B)** Evaluation of the acoustic environment.

The results of the formal questionnaire survey showed that among the four factors that exert an impact on respondents’ perception of Buddhist temples, i.e., the auditory environment, the visual environment, temperature, and humidity. As shown in Figure 1A, 269 people (53.91%) chose the auditory environment as the first influencing factor, 188 people (37.68%) chose the visual environment as the first influencing factor, and fewer than 50 people chose temperature or humidity as the first influencing factor, indicating that within the physical environment of temples, people paid more attention to the acoustic and visual environments.

In terms of respondents’ evaluation of the quietness of the temple acoustic environment, Figure 1B shows that 129 people (25.85%) chose “quiet,” 227 (45.49%) chose “somewhat quiet,” 97 (19.44%) chose “neither quiet nor noisy,” and 46 (9.22%) chose “somewhat noisy” or “noisy.” In terms of the comfort of the acoustic environment of temples, 127 people (25.45%) chose “comfortable,” 255 (51.10%) chose “somewhat comfortable,” 76 (15.23%) chose “no specific feeling,” and 41 (8.22%) chose “somewhat uncomfortable” or “uncomfortable.” In terms of the harmony of the acoustic environment, 132 people (26.45%) chose “harmonious” (i.e., they thought that the acoustic environment of temples was in harmony with the temple atmosphere), 233 (46.69%) chose “somewhat harmonious,” 94 (18.84%) chose “no specific feeling,” and 40 (8.02%) chose “somewhat inharmonious” or “inharmonious.”

**FIGURE 2:**
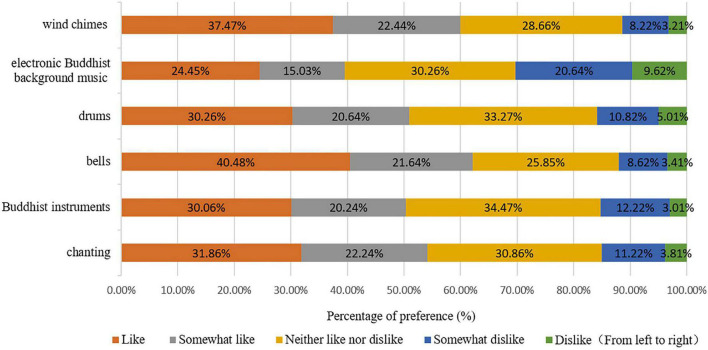
Preferences for the six selected typical sounds in the temples.

The respondents’ preferences for the six selected typical sounds in the temples are shown in Figure 2. Among all kinds of sounds, the proportion of respondents who liked bells was the highest (40.48%), followed by wind chimes (37.47%) and chanting (31.86%). Background electronic Buddhist music (9.62%), followed by drums (5.01%) and chanting (3.81%), were the sounds that respondents most disliked.

The average mental health score of all respondents was 36.69, which indicates a poor level in general. Among the four mental health factors, the overall average score for nervousness was 7.12 (total score was 10), the overall average score for restlessness was 7.53, the overall average score for fatigue was 6.90, and the overall average score for negative affect was 15.13 (the total score was 20, and the equivalent score was 7.57 due to the differing number of items). Fatigue was the lowest scoring of the four factors, indicating that people are facing great stress in current society and that the resulting fatigue has become the most prominent problem affecting mental health.

**FIGURE 3:**
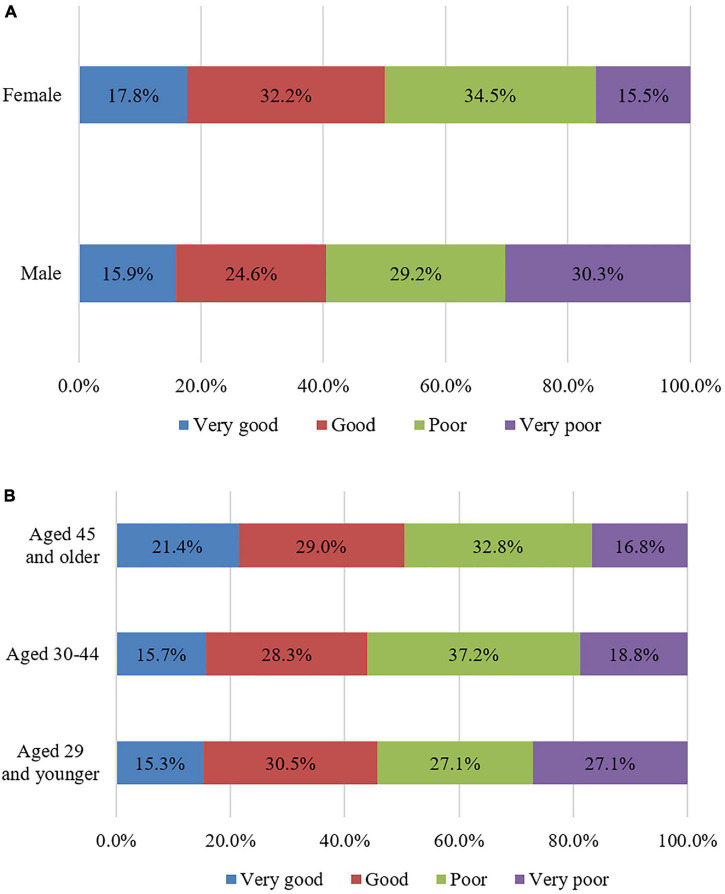
The proportions of respondents’ various mental health states by gender and age. **(A)** Gender; **(B)** Age.

In terms of gender, the mental health score for men was 35.03 and that for women was 37.75. The mean difference in mental health scores between men and women was 2.72, and both scores could be considered to indicate a poor mental health state. An independent-samples *t*-test was performed and indicated a significant difference in mental health between men and women. Figure 3A shows that the proportions of male respondents with “very good,” “good,” and “poor” mental health states were slightly lower than those of female respondents, while the proportion of male respondents with a “very poor” mental health state was 30.3%, almost twice that of female respondents (15.5%). The proportion of male respondents with “poor” and “very poor” mental health states accounted for nearly 60% of the total male respondents, indicating that more female respondents were in good mental states than male respondents. In conclusion, although the mean difference of mental health scores for men and women is not too large, the differences between them are mainly reflected in the proportion of various states.

In terms of age, the mental health score for adolescents (29 years of age and younger) was 36.10, that for middle-aged people (30–44 years of age) was 36.39 and that for middle-aged and elderly people (45 years of age and older) was 37.92. The correlation analysis between respondents’ age and mental health score indicated a significant positive correlation between age and mental health, with a correlation coefficient of 0.099*. This value indicates that the degree of interaction between the two variables is not high, but there is a significant correlation between them (In this paper, * indicates that the correlation between the two variables is significant, that is, *p* < 0.05; ^**^ indicates that the correlation is highly significant, that is, *p* < 0.01). Figure 3B shows that with increasing age, the proportion of people with a “very good” mental health state gradually increases and that the proportion of people with a “very poor” mental health state gradually decreases. This may be because people aged 60 and older face less stress in life or work than adolescents and middle-aged people and thus exhibit better mental health.

Among respondents with different education levels, those with a junior high school education or below and those with a master’s degree or above had high mental health scores, i.e., 37.68 and 37.65, respectively, while those with a college degree had the lowest scores, i.e., 35.90. There was no correlation between mental health score and education level. Among respondents with different occupations, the mental health score was 37.14 for teachers, 36.37 for students, 35.85 for technicians, 35.82 for service personnel, 34.66 for workers and 34.30 for management personnel. Occupation and mental health were significantly correlated (correlation coefficient of 0.284*), mainly manifesting in two factors, nervousness (0.184*) and restlessness (0.182*); the other two factors showed no correlations.

Regarding attitudes toward Buddhist thought, the mental health score was 34.77 for respondents who fully believe in Buddhist thought, 37.66 for those who partially believe in Buddhist thought and 32.88 for those who have little or no faith in Buddhist thought. Regarding the annual number of visits to Buddhist temples, the mental health score was 33.30 for respondents who visited Buddhist temples twice or less per year, 38.76 for those who visited three to five times per year and 43.76 for those who visited more than five times per year. The attitudes toward Buddhist thought and the number of visits to Buddhist temples were significantly correlated with the mental health score, with correlation coefficients of 0.106* and 0.465^**^, respectively. There was no correlation between the respondents’ purpose for visiting Buddhist temples and mental health scores. The mental health score was 36.80 for respondents who were only tourists, 36.36 for those who worship Buddha, and 36.85 for those who visited temples neither to worship Buddha nor as tourists.

We used ANOVA to examine the relationship between mental health score and various religious factors (including the frequency of visiting temples, purpose for visiting temples and attitudes toward Buddhist thought). The test for homogeneity of variance showed that the frequency of visiting temples and purpose for visiting temples were suitable for ANOVA. However, the variance of attitudes toward Buddhist thought was heterogeneous, so ANOVA could not be used. The ANOVA results show that there is a significant difference between the mental health scores of each visit frequency (the *p*-values of single factor analysis and multiple analysis are all less than 0.001); however, there is no significant difference between the mental health scores of various visit purposes (the *p*-values of single factor analysis and multiple analysis are all greater than 0.600).

### The Relationship Between Han Buddhist Temple Soundscapes and Mental Health

Previous studies have shown that the acoustic environment of religious sites may exert an impact on mental health. In our questionnaire, the overall acoustic environment of temples was evaluated by respondents based on the three aspects of quietness, comfort and harmony; respondents’ mental health scores based on their evaluations of the acoustic environment are shown in Table 3. The results showed that the respondents who assessed the temple as quiet or somewhat quiet (comfortable or somewhat comfortable, harmonious or somewhat harmonious) had a significantly better mental health status than those who assessed the temple as noisy or somewhat noisy (somewhat uncomfortable or uncomfortable, somewhat inharmonious or inharmonious).

**TABLE 3:** The mental health scores based on their evaluations of the acoustic environment.

Type	Mental health scores			
	Quiet (comfortable/harmonious)	Somewhat quiet (somewhat comfortable/somewhat harmonious)	Neither quiet nor noisy (no specific feeling/no specific feeling)	Somewhat noisy or noisy (somewhat uncomfortable or uncomfortable/somewhat inharmonious or inharmonious)
Quietness	37.03	37.16	36.64	33.50
Comfort	36.42	38.34	34.47	31.37
Harmony	37.95	37.95	34.91	29.35

Respondents’ evaluations of these three acoustic environmental factors were all significantly correlated with the mental health score; the correlation coefficients for quietness, comfort and harmony were 0.102*, 0.113*, and 0.213^**^, respectively. Respondents’ evaluation of the harmony of the acoustic environment had the highest correlation with mental health and was significantly correlated with the four mental health factors (nervousness, restlessness, fatigue, and negative affect). Quietness was only significantly correlated with nervousness and fatigue, while comfort was significantly correlated with restlessness, fatigue, and negative affect.

Among the six sounds typical in a Han Chinese Buddhist temple, preferences for three sounds were correlated with respondents’ mental health: wind chimes (0.127^**^), bells (0.120^**^), and chanting (0.094*). The three non-correlated sound preferences were Buddhist instruments, drums and electronic Buddhist background music. Among the sound preferences that were correlated with mental health, the correlation between the preference for wind chimes and mental health was the highest, and the preference for wind chimes was significantly correlated with four mental health factors (nervousness, restlessness, fatigue, and negative affect). Preferences for bells and for chanting were significantly correlated only with fatigue and negative affect.

### Effect of Religious Belief-Related Factors on the Relationship Between Respondents’ Evaluations of the Overall Acoustic Environment of Han Chinese Buddhist Temples and Their Mental Health

#### Moderating Effect of Religious Belief-Related Factors

**FIGURE 4:**
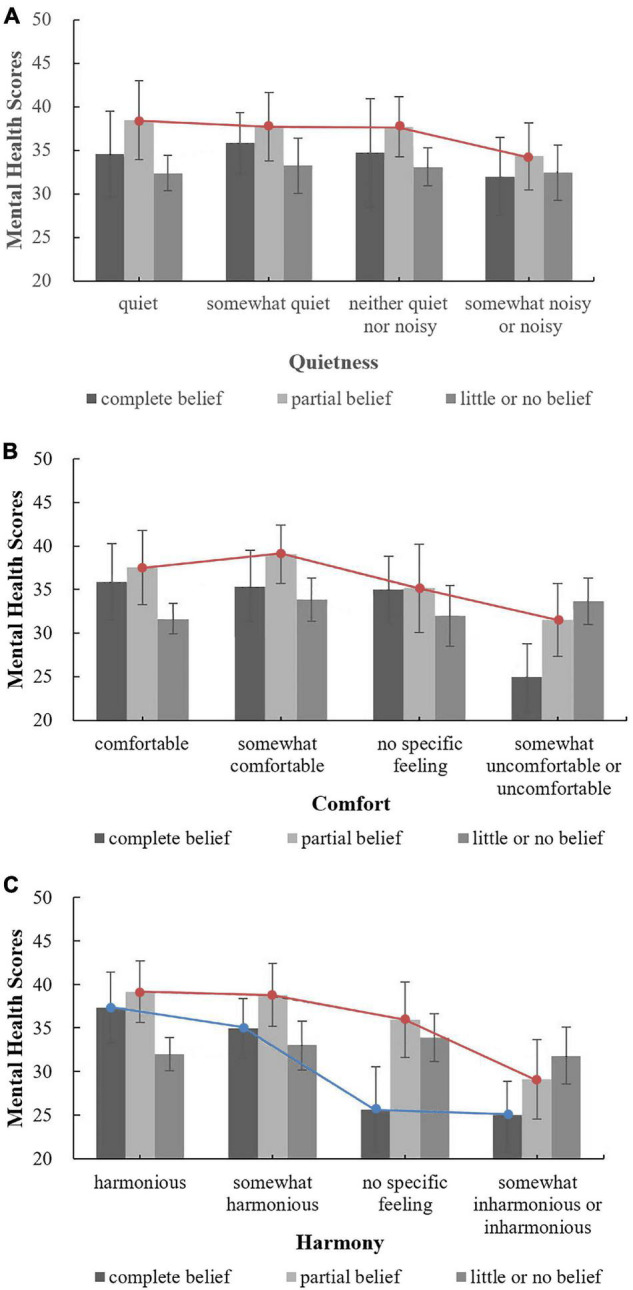
The relationships between the evaluations of the overall acoustic environment and mental health by level of belief in Buddhist thought. **(A)** Quietness; **(B)** Comfort; **(C)** Harmony.

**FIGURE 5:**
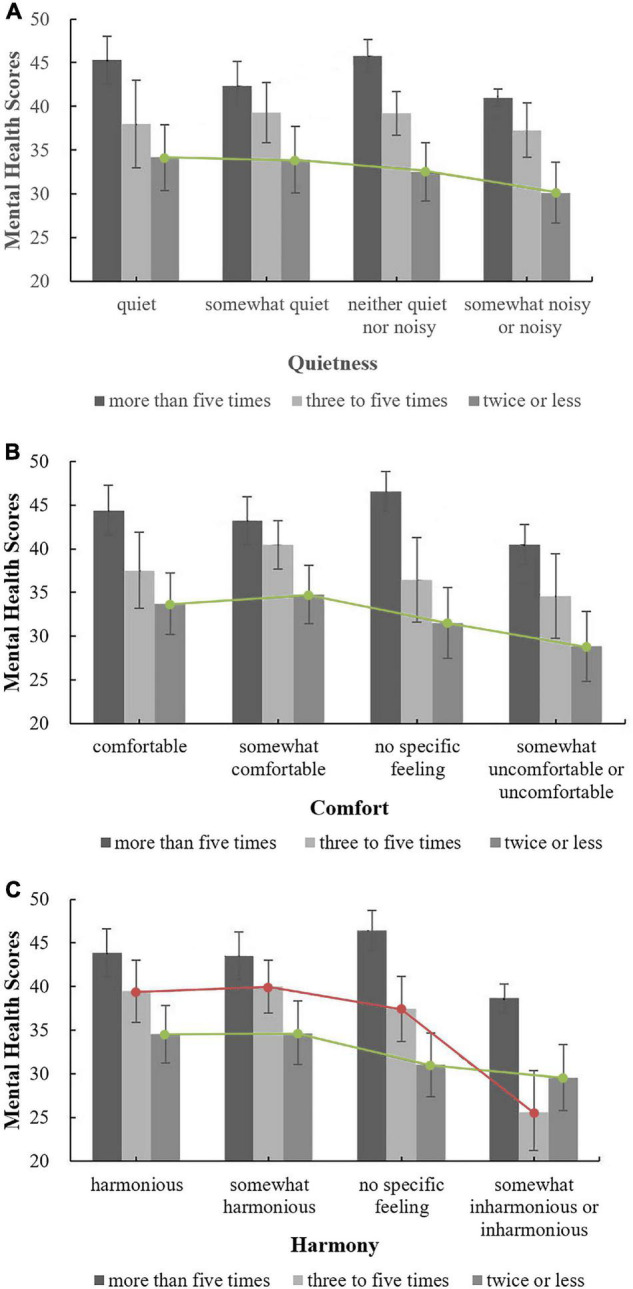
The relationships between the evaluations of the overall acoustic environment and mental health under different frequencies. **(A)** Quietness; **(B)** Comfort; **(C)** Harmony.

**FIGURE 6:**
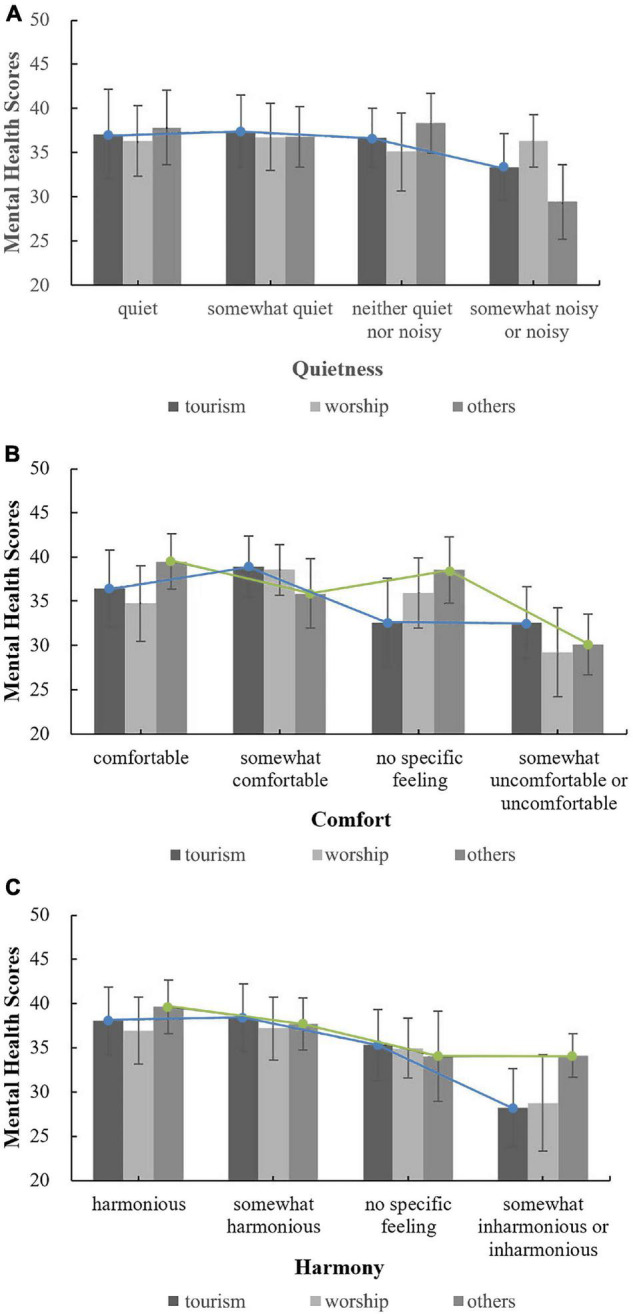
The relationships between the evaluations of the overall acoustic environment and mental health by purpose of visiting. **(A)** Quietness; **(B)** Comfort; **(C)** Harmony.

**FIGURE 7:**
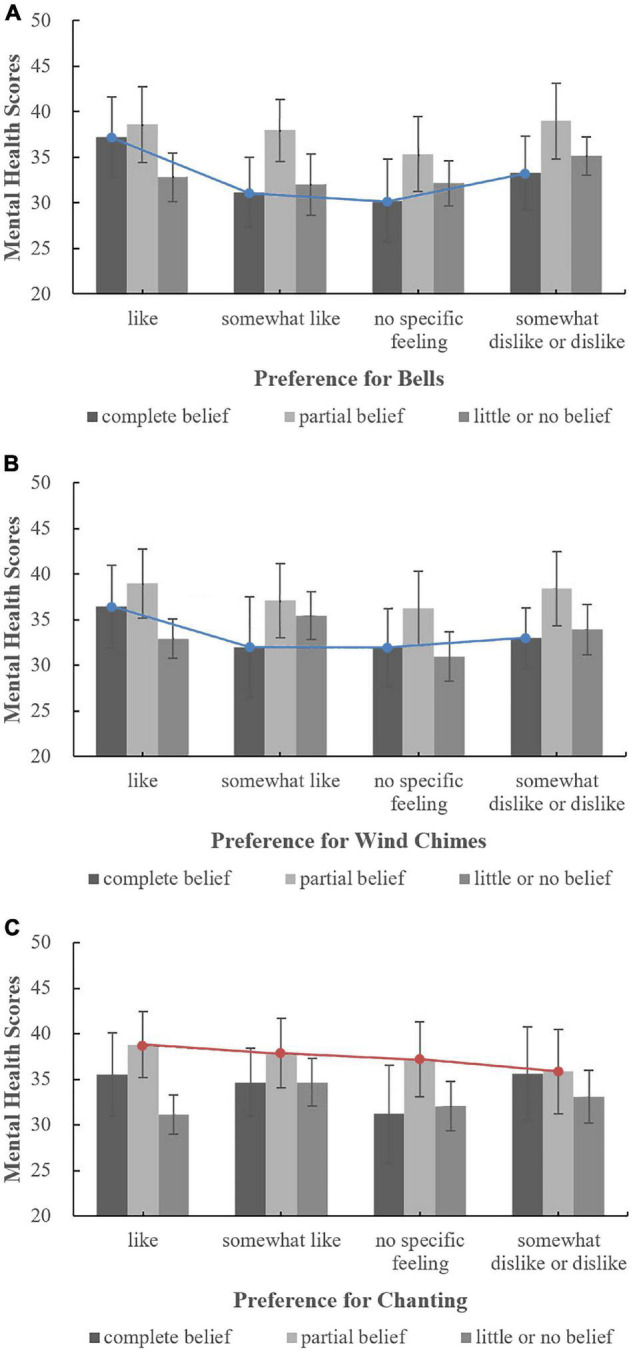
The relationships between sound preferences and mental health by level of belief in Buddhist thought. **(A)** Bells; **(B)** Wind chimes; **(C)** Chanting.

**FIGURE 8:**
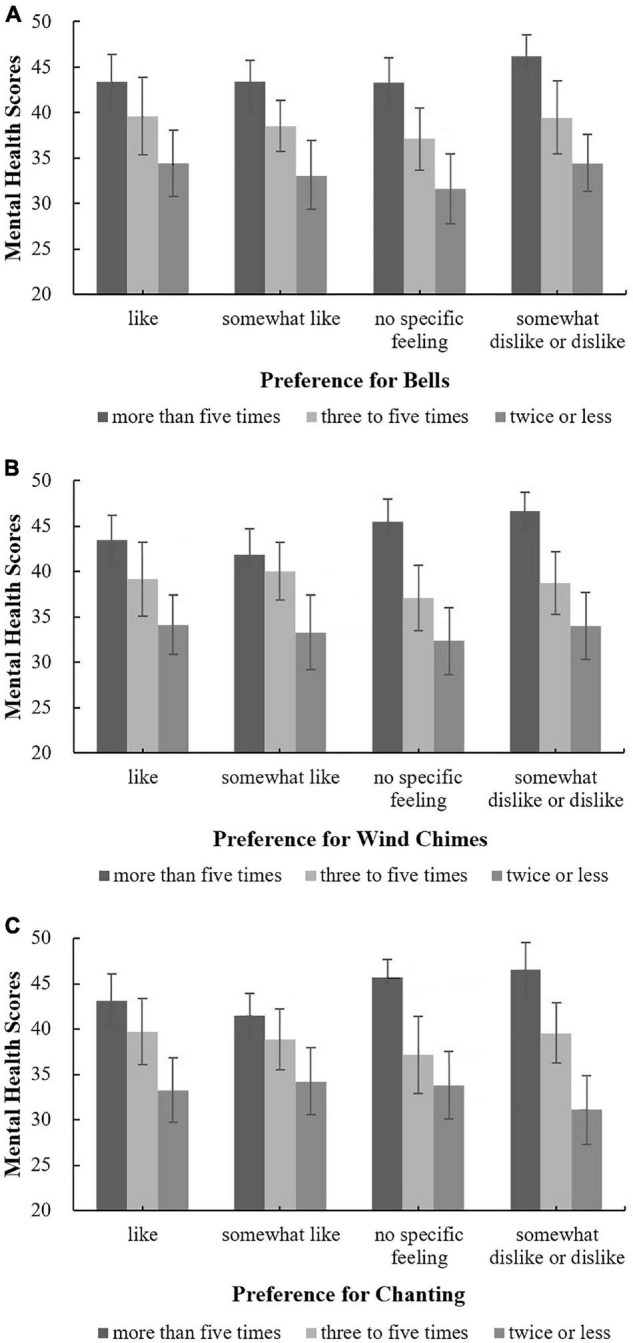
The relationships between sound preferences and mental health under different frequencies. **(A)** Bells; **(B)** Wind chimes; **(C)** Chanting.

**FIGURE 9:**
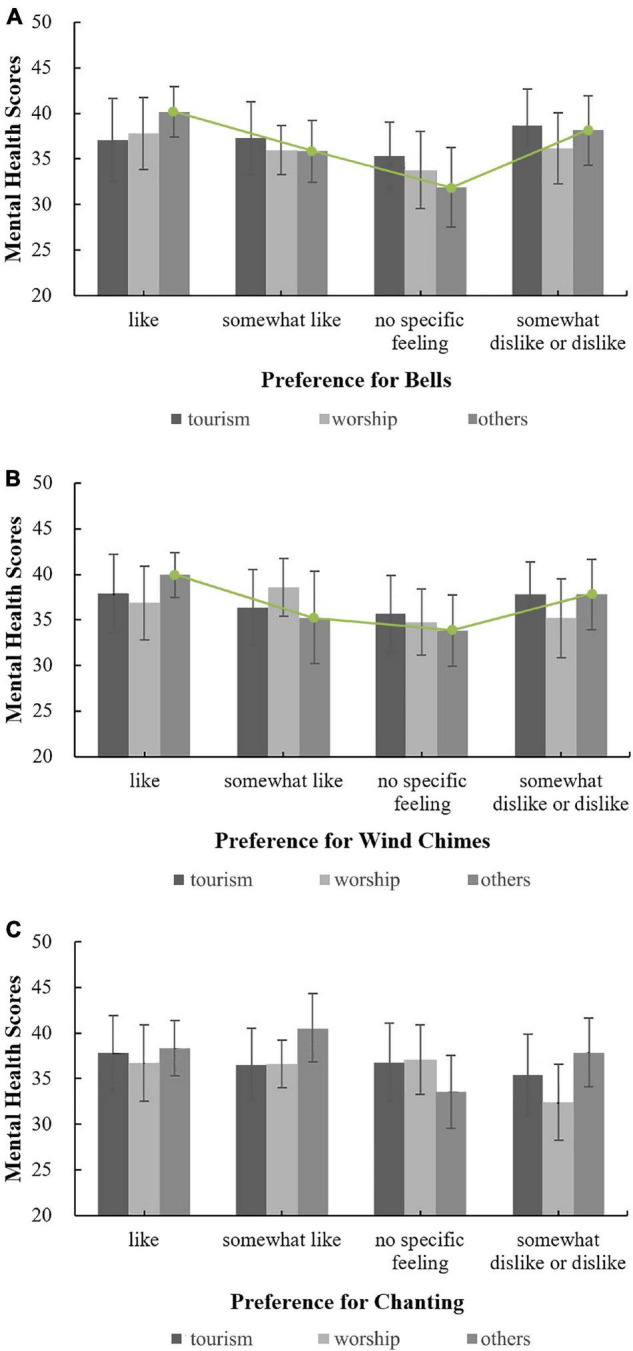
The relationships between sound preferences and mental health by purpose for visiting. **(A)** Bells; **(B)** Wind chimes; **(C)** Chanting.

The impact of respondents’ belief-related factors on the correlation among respondents’ evaluations of three acoustic environmental factors and their mental health was analysed. Figure 4 shows the relationships between the evaluations of the overall acoustic environment and mental health by level of belief in Buddhist thought (Note: In Figures 4–9 in this paper, only when the correlations were significant were the lines connected to the means added). For those who fully or partially believe in Buddhist thought (those with partial beliefs accounted for the largest proportion of the respondents), there was an approximately linear negative association between mental health and evaluations of the overall acoustic environment of temples. However, for people who have little or no belief in Buddhist thought, there was no such linear association between mental health and the evaluations of the overall acoustic environment of temples. In general, as shown in Figures 4A–C, for those respondents who evaluated the temple as quiet (comfortable, harmonious) or somewhat quiet (somewhat comfortable, somewhat harmonious), the order of mental health scores, ranked from high to low, was relatively regular, i.e., partial belief > complete belief > little or no belief. In contrast, for respondents who indicated no feelings or chose somewhat noisy or noisy (somewhat uncomfortable or uncomfortable, somewhat inharmonious or inharmonious), there was no such pattern. These results may indicate that a good acoustic environment in a temple can regularly and positively affect mental health and that if the acoustic environment in a temple prompts negative or no feelings, the impact on people’s mental health is not obvious or is irregular. Overall, these findings indicate the important role of a good acoustic environment for people who visit temples. Correlation analysis showed that for people who partially believe in Buddhist thought, as shown in Figures 4A–C, respondent evaluations of the three acoustic environmental factors were all correlated with mental health; the correlation coefficients for quietness, comfort and harmony were 0.125*, 0.130*, and 0.226^**^, respectively. In addition, for those who fully believe in Buddhist thought, as shown in Figure 4C, only the evaluation of the harmony of the acoustic environment in the temple was significantly correlated with mental health, with a correlation coefficient of 0.360^**^. These findings indicate that, compared with evaluations of quietness or comfort, evaluations of harmony were correlated with mental health in respondents with more varied levels of belief in Buddhist thought (including those who fully believe in Buddhist thought). Moreover, the correlation coefficient was larger, and the degree of correlation was more significant (from *p* < 0.05 to *p* < 0.01). As shown in Figures 4A–C, there was no correlation between respondents’ evaluations of the acoustic environment of temples and mental health among those who had little or no belief in Buddhist thought.

Regarding the annual frequency of Buddhist temple visitation, the relationships between evaluations of the acoustic environment and mental health under different frequencies are shown in Figure 5. The respondents who visited Buddhist temples more than five times per year and indicated no specific feelings regarding the quietness, comfort and harmony of the acoustic environment of temples had the highest mental health scores, which indicates that people who visited temples frequently (who may be Buddhist believers) may not have special requirements for the acoustic environment of temples and that their mental health state is high and not affected by the acoustic environment (i.e., they might already be accustomed to the acoustic environment of temples). However, for those who visit temples twice or less per year, one possible way to improve their mental health is to increase the number of visits to the temple by improving the overall acoustic environment of temples. Correlation analysis showed that for people who visited Buddhist temples twice or less per year, as shown in Figures 5A–C, respondent evaluations of the three acoustic environmental factors were all correlated with mental health; the correlation coefficients for quietness, comfort and harmony were 0.155*, 0.166*, and 0.223^**^, respectively. In addition, as shown in Figure 5C, for people who visit Buddhist temples three to five times per year, respondents’ evaluation of the harmony of the acoustic environment was significantly correlated with mental health, with a correlation coefficient of 0.177^**^. For other conditions, there was no correlation. These findings indicate that, compared with evaluations of quietness or comfort, evaluations of harmony were correlated with mental health in respondents with more varied frequencies of visitation (including those who visit Buddhist temples three to five times per year).

Regarding the purpose of temple visitation, Figure 6 shows the relationships between respondents’ evaluations of the overall acoustic environment and mental health by purpose of visiting. These results indicated that, as shown in Figures 6A,C, for people who are only tourists, when the acoustic environment of temples changes from quiet (harmonious) to noisy (inharmonious), the mental health score decreases approximately linearly. However, this trend was not observed for those who worship Buddha or those who visit for purposes other than worship or tourism. For people with different purposes for visiting, when the acoustic environment of temples was evaluated as quiet or somewhat quiet (harmonious or somewhat harmonious), the mental health scores were very close, but when the acoustic environment of temples was evaluated as somewhat noisy or noisy (somewhat inharmonious or inharmonious), the mental health scores differed greatly, indicating that the design of the acoustic environment of temples should consider the various purposes for temple visitation to improve people’s mental health. Correlation analysis showed that for tourists, respondents’ evaluations of all three acoustic environmental factors were correlated with mental health; the correlation coefficients for quietness, comfort and harmony were 0.138*, 0.168^**^, and 0.247^**^, respectively. For those who worship Buddha, as shown in Figures 6A–C, respondents’ evaluations of all three acoustic environmental factors were not correlated with mental health, which may indicate that, compared with tourists, people who worship Buddha are more adaptable to the acoustic environment of temples or are not affected by the acoustic environment. For people who visit for purposes other than worship or tourism, as shown in Figures 6B,C, respondents’ evaluations of the comfort and harmony of the acoustic environment in the temple were correlated with mental health; the correlation coefficients were 0.211* and 0.274^**^, respectively. Among the three acoustic environmental factors, for various groups of people, evaluations reflecting harmony had the highest correlation with mental health.

In summary, the results of this study showed that for respondents with a partial belief in Buddhist thought, respondents who visited temples twice or less per year or those who visited temples only for tourism purposes, the influence of religious belief-related factors on the correlation between respondents’ evaluations of the overall acoustic environment of temples and mental health was more significant.

#### Influence of Demographic Factors

Respondents’ demographic factors also affected the correlation between their evaluations of the overall acoustic environment of temples and their mental health. For gender, the analysis showed an approximately linear, downward trend for women’s mental health scores when evaluations of the acoustic environment of temples changed from quiet (harmonious) to noisy (inharmonious); no such trend was observed for men. Correlation analysis showed that for women, evaluations of the three acoustic environmental factors were all significantly correlated with mental health, but for men, there were no such correlations. Regarding age, for adolescents (below 29 years of age), evaluations indicating comfort and harmony were correlated with mental health; for middle-aged people (30–44 years of age), evaluations indicating quietness and harmony were correlated with mental health; and for middle-aged and elderly people (older than 45 years of age), there were no correlations between evaluations of the three acoustic environmental factors and mental health. Regarding education level, for people with a junior high school education or below, evaluations indicating comfort and harmony were correlated with mental health, and for people with a college degree, evaluations indicating harmony were correlated with mental health. In terms of occupations, for service personnel, evaluations indicating comfort and harmony were correlated with mental health, and for students and teachers, evaluations indicating harmony were correlated with mental health; there were no correlations for other occupations. Previous studies have similarly shown that gender, age, education, and other factors can affect the correlation between the acoustic environment and human mental health (Niemann et al., 2006; Van Kempen et al., 2011; Anilan, 2014), indicating that the relationship between the acoustic environment of both Eastern and Western religious sites and mental health may be affected by factors such as gender, age, and education.

#### Influence of Demographic Factors on the Moderating Effect of Religious Belief-Related Factors

One of the research aims of this paper is to determine the influence of religious belief-related factors on the correlation between respondents’ evaluations of the acoustic environment of temples and their mental health. However, can demographic factors, such as gender, age, and education level, affect this moderation? The relationships between human demographic factors and religious belief-related factors were analysed. The results showed that gender and religious belief-related factors were not correlated and that gender did not affect the moderating effect of religious belief-related factors. Regarding the attitudes toward Buddhist thought, frequency of temple visitation and purpose for visiting, the correlation coefficients for age were 0.146^**^, 0.151^**^, and 0.137^**^, respectively; those for education level were −0.133^**^, −0.022/0.620, and 0.165^**^, respectively; and those for occupation were 0.186/0.124, 0.156/0.552, and 0.224^**^, respectively. These coefficients indicted that the overall correlation was not high. Additionally, the population 30–44 years of age, the population with a college degree and the student population (collectively accounting for the highest proportion of respondents) were analysed. The results showed that the moderating effect of religious belief-related factors in these populations was not significantly different from that for the whole population; therefore, human demographic factors did not significantly affect the moderating effect of religious belief-related factors on the correlation between respondents’ evaluations of the temple acoustic environment and their mental health.

### Effect of Religious Belief-Related Factors on the Relationship Between Preference for Sounds in Han Chinese Buddhist Temples and Mental Health

#### Moderating Effect of Religious Belief-Related Factors

The moderating effect of respondents’ belief-related factors on the correlation between three sound preferences (bells, wind chimes, and chanting) and mental health was analysed, and the influence of attitudes toward Buddhist thought on the relationships between sound preferences (bells, wind chimes, and chanting) and mental health was investigated, as shown in Figure 7. Among respondents who enjoyed the three sounds, the mental health scores corresponding to different attitudes toward Buddhist thought were ordered as follows: partial belief > complete belief > little or no belief. However, among respondents who did not indicate that they liked the three sounds, the mental health scores associated with different attitudes toward Buddhist thought did not exhibit the same pattern, a finding similar to the relationship between respondents’ evaluations of the overall acoustic environment and mental health analysed in the previous section. These results suggest that the sounds in temples that people enjoy may regularly affect their mental health, whereas sounds that people dislike or about which they have no feelings are unlikely to regularly affect their mental health. Correlation analysis showed that, as shown in Figures 7A,B, for people who fully believe in Buddhist thought, preferences for bells and wind chimes were both correlated with mental health; the correlation coefficients were 0.366^**^ and 0.297*, respectively. As shown in Figure 7C, for those who partially believe in Buddhist thought, the preference for chanting was correlated with mental health; the correlation coefficient was 0.104*. There were no correlations for other attitudes toward Buddhist thought. Overall, for people who fully believe in Buddhist thought, the correlation between the preference for bells and mental health was highest, a finding that is consistent with the results of one of our previous studies, i.e., the sound of bells is generally considered to be the most representative sound in Han Chinese Buddhist temples (Zhang et al., 2018).

The effect of annual visit frequency on the relationship between preferences for the sounds of bells, wind chimes and chanting and mental health was analysed; the results are shown in Figures 8A–C. For people with different visit frequencies, there was no correlation between the preferences for various temple sounds and mental health, indicating that visit frequency did not have a moderating effect on the relationship between sound preference and mental health. Thus, to improve the mental health of people who visit temples, it may not be necessary to group people based on visit frequency, i.e., people with different visit frequencies can be treated as a whole population.

The effect of the purpose of visiting Buddhist temples on the relationship between mental health and preferences for bells, wind chimes and chanting was analysed; the results are shown in Figure 9. For people with purposes other than worshipping Buddha and tourism, as shown in Figures 9A,B, when their preferences for bells and wind chimes changed from “like” to “no specific feelings,” the mental health score linearly declined, an association that was not reflected in the preferences for chanting, as shown in Figure 9C. For those with purposes other than worshipping Buddha or tourism, the influence of sound preferences on mental health was irregular. Therefore, when designing and choosing sounds in temples, people’s auditory preferences should be comprehensively considered independent of their purpose for visiting. Correlation analysis showed, per Figures 9A,B, that for people whose purpose for visiting was neither worship nor tourism, there was a correlation between preferences for bells and wind chimes and mental health; the correlation coefficients were 0.260^**^ and 0.255*, respectively. There were no correlations between sound preferences and mental health under other conditions.

In summary, for people who fully believe in Buddhist thought or whose purpose for visiting temples is neither for worship nor for tourism, their religious belief-related factors exert a more significant impact on the correlation between sound preferences in temples and mental health than for people without these religious characteristics.

#### Influence of Demographic Factors

For women, preferences for the three sounds were correlated with mental health, but no such correlation was observed for men. The effect of age on the relationship between mental health and preferences for bells, wind chimes and chanting was analysed. When evaluating the preferences for bells and chanting, the mental health scores corresponding to each preference among adolescents were the same as those among middle-aged people but were substantially different from those among people aged 45 and older. Among the people who selected “somewhat dislike” or “dislike” for the three sounds of bells, wind chimes and chanting, those aged 45 and older had the best mental health. This finding may indicate that people over 45 years of age have a higher tolerance of various sounds in temples and that their mental health is unaffected by sounds that they dislike. Correlation analysis showed that adolescents’ preferences for bells and wind chimes were correlated with mental health; the correlation coefficients were 0.217^**^ and 0.163*, respectively. Middle-aged people’s preference for bells was correlated with mental health, with a correlation coefficient of 0.147*. The preferences of respondents aged 45 and older for the three sounds were not correlated with mental health. In terms of education level, for the respondents with a college degree, there were correlations between preferences for bells and wind chimes and mental health; the correlation coefficients were 0.178^**^ and 0.169^**^, respectively. There were no correlations between sound preferences and mental health among people with other education levels. Regarding occupations, only workers’ preference for chanting and students’ preference for bells were correlated with mental health; there were no correlations for other occupations. In summary, the gender, age, education, and occupation of the respondents affected the correlation between the preferences of sounds in temples and mental health to a certain extent.

#### Influence of Demographic Factors on the Moderating Effect of Religious Belief-Related Factors

In section “Effect of Religious Belief-Related Factors on the Relationship Between Respondents’ Evaluations of the Overall Acoustic Environment of Han Chinese Buddhist Temples and Their Mental Health,” the results showed that there is no correlation between gender and religious belief-related factors, and the correlations between belief-related factors and age, education and occupation were not high. Additionally, this study analysed the population aged 30–44, the population with a college degree and the student population (collectively accounting for the highest proportion of respondents), with the results showing that the moderating effect of religious belief-related factors on sound preferences in temples and mental health in these populations was basically consistent with that for the population as a whole. Therefore, respondents’ demographic factors did not significantly affect the moderating effect of religious belief-related factors on the correlation between sound preferences in temples and mental health.

## Discussion

### Comparison of Respondents’ Evaluations of the Overall Acoustic Environment and Sound Preferences

The moderating effect of belief-related factors on the correlation between respondents’ evaluations of the overall acoustic environment and their mental health was compared with the effect of belief-related factors on the correlation between sound preferences and mental health, illustrating that, in terms of the attitudes toward Buddhist thought, people who partially believe in Buddhist thought accounted for the highest proportion of respondents. For these respondents, their evaluations of the three acoustic environmental factors were correlated with mental health; however, only a preference for chanting was correlated with mental health, with a correlation coefficient less than that for any of the evaluations of acoustic environmental factors, indicating that the impact of the evaluations of the overall acoustic environment on mental health is more important than a single sound. In terms of the frequency of Buddhist temple visits, the population with two or fewer annual visits to Buddhist temples accounted for the highest proportion of respondents, and their evaluations of the three acoustic environmental factors were correlated with their mental health. However, there was no correlation between sound preferences and mental health among people with different visit frequencies. Regarding the purpose of visiting Buddhist temples, tourists accounted for the highest proportion of respondents, and their evaluations of acoustic environmental factors were correlated with their mental health, while there was no correlation between sound preferences and mental health for this group of people. These results indicate that the influence of religious belief-related factors on the correlation between the evaluations of the overall acoustic environment and mental health is significantly stronger than the influence of religious belief-related factors on the correlation between sound preferences and mental health.

### Comparison With Related Studies

Religions in China mainly include Buddhism, Taoism, and Islam. There are few studies on the relationship between the acoustic environment or soundscape in Chinese religious places and mental health. We compare the results of this research with similar studies in other countries on the relationship between Islamic or Christian soundscapes and mental health. According to several studies on Islamic religious music, the positive impact of religious music on mental health is undeniable, and in Islamic countries, the voice of the holy Quran is used to heal patients (Heidari and Shahbazi, 2013; Babaii et al., 2015; Jafari et al., 2016).

Compared with the soundscapes or sound environment in mosques, the soundscapes in Han Chinese Buddhist temples do not seem to have such a strong impact on human mental health, which is reflected in the low correlation coefficient. This may also be related to the Chinese religious conception of Han Chinese Buddhism. It is generally believed that most Chinese people recognise Buddhist thought, but their beliefs are not very pious. Interestingly, the results of this paper are similar to the results of a study on the relationship between American Christian music and the mental health of elderly individuals, which showed that the correlations between the frequency of listening to religious music in late life and several different aspects of mental health (death anxiety, life satisfaction, self-esteem, and sense of control) were salutary and statistically significant but relatively weak, and the correlations ranged from 0.076 to 0.171 in magnitude (Bradshaw et al., 2015).

### Application of the Results

The value of this study is mainly reflected in three aspects. First, the results of this study show that a good acoustic environment and pleasant sounds in temples can regularly and positively affect the mental health of people who visit temples, indicating the important role of the acoustic environment of temples in human mental health. Related studies have also shown that good soundscapes could help Chinese people enjoy positive environmental experiences and relieve stress (Zhang et al., 2017). Therefore, when designing temples, it is necessary to fully consider the incorporation of Buddhism-related sounds and the creation of a religious atmosphere in the acoustic environment. Considering all kinds of soundscape evaluation conditions, the mental health of people who visit Buddhist temples more often is better than that of people who visit the temple less often. Therefore, if we can create a good acoustic environment to attract people to visit the temple more often, it may achieve the purpose of improving people’s mental health. Second, various religious belief-related factors affect the relationship between respondents’ evaluations of the acoustic environment of or sound preferences in temples and their mental health to varying degrees; therefore, when designing the sound environment of temples, belief-related factors should be considered. Reasonable zoning should be implemented based on the characteristics of different populations so that a suitable acoustic environment or pleasant sounds can be created for people with different religious characteristics. Even for people who are not sensitive to religious acoustic environments, their mental health can improve by hearing sounds they enjoy. In addition, the results of this research show that the psychological feelings and tolerance of the temple soundscape between elderly individuals and the young or middle-aged are not the same, and mental health of elderly individuals is not related to his evaluation of the acoustic environment or preference for various sounds in the temples. Therefore, the acoustic environment could be designed according to the age characteristics of the visitors in the temple; for example, more consideration can be given to the auditory needs of young or middle-aged people. Third, this study provides some specific measures for the design of the acoustic environment of temples. For example, the results show that among temple sounds, bells, and wind chimes are most closely related to the mental health of people with various religious beliefs. Therefore, special attention should be devoted to the distribution of these two sounds in the acoustic environment of temples. The results of this study also show that harmony, one aspect of the acoustic environment in temples, has the highest correlation with the mental health of various groups of people and that harmony is correlated with all four mental health factors explored in this study. Therefore, in the design of the acoustic environment of temples, the most important consideration is whether the sound environment is harmonious with the religious atmosphere of the temple. In addition, the results of this study can also provide a reference for the acoustic environmental design of other similar religious sites.

### Research Limitations

The method used in this study was a questionnaire survey. Therefore, the results regarding the relationship between mental health and personal evaluations of soundscapes need to be verified by other methods. The study subjects were tourists and believers in Han Chinese Buddhism; Buddhist monks were not included. Therefore, the types of research subjects need to be expanded in future studies. This study focused on Han Chinese Buddhism, and no research or comparative analysis was conducted for other religions in China (such as Taoism and Tibetan Buddhism). The correlations between mental health and soundscapes in other religious places may have similarities to and differences from those revealed in this study. Whether these similarities are related to the national characteristics of Chinese people and whether the differences are related to various religious doctrines and rituals require further analysis.

In addition, this research did not include the acoustic parameters of soundscapes in Buddhist temples. In our previous research on soundscapes in Han Chinese Buddhist temples, we analysed acoustic parameters such as sound pressure level, loudness, and sharpness. The results showed that the acoustic parameters of soundscapes in Han Chinese Buddhist temples could affect the feelings of respondents (Zhang et al., 2016, 2018). In future research, we plan to combine acoustic parameters with subjective health evaluations, which are believed to provide better guidance for the soundscape design of temples.

## Conclusion

As the religion with the highest number of believers in the world’s most populous country, Han Chinese Buddhism has played an important role in the development of Chinese society and the lives of Chinese people. Soundscapes are important means of creating a religious atmosphere in Han Chinese Buddhist temples. However, the influence of soundscapes on Chinese people’s mental health has not received due attention from scholars. This study used a questionnaire survey to analyse the correlation between Chinese people’s evaluations of Buddhist temple soundscapes (including the overall acoustic environment of the temple and sound preferences) and mental health as well as the influence of respondents’ religious belief-related factors on this correlation. After 499 valid questionnaires were analysed, the following main conclusions were obtained.

(1) There were significant correlations between the evaluations of the quietness, comfort and harmony of the acoustic environment of Buddhist temples and respondents’ mental health scores, with correlation coefficients ranging from 0.10 to 0.22. Among the religion-related sounds in temples, bells, wind chimes and chanting were significantly correlated with respondents’ mental health, with correlation coefficients ranging from 0.10 to 0.13. The correlation between the evaluations of the overall acoustic environment in temples and mental health was generally higher than that between sound preferences and mental health, and the correlation between an acoustic environment evaluated as harmonious and mental health was the highest.
(2) Respondents’ belief-related factors, including their attitudes toward Buddhist thought, annual number of visits to temples and their purposes for visiting temples, all exerted a significant impact on the relationship between their evaluations of the overall acoustic environment of temples and mental health. For people who fully believe in Buddhist thought or those who visit Buddhist temples three to five times per year, there was a positive correlation between the evaluations of the acoustic environment as harmonious and mental health. For people who partially believe in Buddhist thought, people who visit temples twice or less per year, or people who visit temples as tourists, evaluations of all three acoustic environmental factors were positively correlated with mental health, with correlation coefficients ranging from 0.13 to 0.25. Additionally, some demographic factors somewhat affected the relationship between evaluations of the acoustic environment and mental health, but demographic factors did not significantly affect the moderating effect of religious belief-related factors on the correlation between evaluations of the acoustic environment of temples and mental health.
(3) Among the respondents’ belief-related factors, attitudes toward Buddhist thought and purpose for temple visitation exerted a significant impact on the relationship between sound preferences and mental health. For people who partially believe in Buddhist thought, there was a significant correlation between the preference for chanting and mental health, and for people who fully believe in Buddhist thought or those who visit temples neither for worship nor for tourism purposes, there was a significant correlation between the preferences for bells and wind chimes and mental health, with correlation coefficients ranging from 0.26 to 0.37. Some demographic factors affected the relationship between sound preferences and mental health, but the demographic actors did not significantly affect the moderating effect of religious belief-related factors on the correlation between sound preferences in temples and mental health.

## Data Availability Statement

The original contributions presented in the study are included in the article/supplementary material, further inquiries can be directed to the corresponding author/s.

## Ethics Statement

Ethical review and approval was not required for this study in accordance with the local legislation and institutional requirements. Permission for carrying out this work was obtained from the JangHo Architecture College, Northeastern University. The participants were provided written informed consent to participate in the study.

## Author Contributions

DZ: study conception, questionnaire design, and manuscript writing. CK: research idea, questionnaire survey, and statistical analysis of the data. MZ: interpretation of the results, supervision, and project administration. JK: overall supervision and the structuring of the manuscript. All authors reviewed the results and approved the final version of the manuscript.

## Conflict of Interest

The authors declare that the research was conducted in the absence of any commercial or financial relationships that could be construed as a potential conflict of interest.

## Publisher’s Note

All claims expressed in this article are solely those of the authors and do not necessarily represent those of their affiliated organizations, or those of the publisher, the editors and the reviewers. Any product that may be evaluated in this article, or claim that may be made by its manufacturer, is not guaranteed or endorsed by the publisher.
